# Arginase Flavonoid Anti-Leishmanial *in Silico* Inhibitors Flagged against Anti-Targets

**DOI:** 10.3390/molecules21050589

**Published:** 2016-05-05

**Authors:** Sanja Glisic, Milan Sencanski, Vladimir Perovic, Strahinja Stevanovic, Alfonso T. García-Sosa

**Affiliations:** 1 Center for Multidisciplinary Research, Institute of Nuclear Sciences VINCA, University of Belgrade, P.O. Box 522, 11001 Belgrade, Serbia; sanja@vin.bg.ac.rs (S.G.); sencanski@vin.bg.ac.rs (M.S.); vladaper@vinca.rs (V.P.); strahinja.stevanovic@protonmail.com (S.S.); 2 Institute of Chemistry, University of Tartu, Ravila 14a, Tartu 50411, Estonia

**Keywords:** *Leishmania*, arginase, flavonoid, *in silico*, screen, anti-target, leishmaniasis, natural products, pregnane-X-receptor, sulfotransferase, cytochrome P450

## Abstract

Arginase, a drug target for the treatment of leishmaniasis, is involved in the biosynthesis of polyamines. Flavonoids are interesting natural compounds found in many foods and some of them may inhibit this enzyme. The MetIDB database containing 5667 compounds was screened using an EIIP/AQVN filter and 3D QSAR to find the most promising candidate compounds. In addition, these top hits were screened *in silico versus* human arginase and an anti-target battery consisting of cytochromes P450 2a6, 2c9, 3a4, sulfotransferase, and the pregnane-X-receptor in order to flag their possible interactions with these proteins involved in the metabolism of substances. The resulting compounds may have promise to be further developed for the treatment of leishmaniasis.

## 1. Introduction

Leishmaniasis, a vector-borne infectious disease, is endemic in 98 countries in parts of the tropics, subtropics, and in Europe [1,2,3]. Leishmaniasis can have skin, mucocutaneous, and visceral presentations caused by 20 different *Leishmania* parasite species. This is a neglected tropical disease (NTD) and a major public health problem that currently affects 12 million people with estimated 0.2 to 0.4 million of new visceral leishmaniasis cases and 0.7 to 1.2 million of new cutaneous leishmaniasis cases each year, globally [1,4]. It is also a severe co-infection with human immunodeficiency virus infection (HIV), where there is a high chance of developing the full-blown clinical disease with high relapse and mortality rates [5].

The synthesis of polyamines (PA) is essential for intracellular growth of *Leishmania* [6]. Arginase is the first enzyme involved in PA biosynthesis and hydrolyses arginine into ornithine and urea [7]. Blocking arginase can lead to oxidative stress in parasite cells, due to a deficiency in trypanothione production and thus promote infection control [8]. *Leishmania* becomes auxotrophic for polyamines when the arginase gene is deleted, showing the significance of this enzyme for parasite survival [9]. Arginase has two characteristics that are important for a parasite drug target: distinction from the mammalian target [10] and absolute necessity for the survival of the pathogen [11]. Among individual infectious diseases, leishmaniasis causes the ninth largest disease burden, but because of a lack of commercial interest it is still one of the most neglected diseases in terms of drug development [12,13]. There are no vaccines and the control of leishmaniasis primarily depends on chemotherapy. Current chemotherapies against leishmaniasis are highly toxic, cause side effects, and commonly, drug resistance. In addition, compliance of patients to therapy is low as treatment is long and expensive [12].

Development of new effective chemotherapeutic agents for treatment of leishmaniasis is greatly needed. Natural products are a promising source of low toxicity, efficient, and widely accessible drug candidates [14]. In addition and very importantly, natural products are a rich source of compounds with anti-leishmanial activity [15]. Previously, it was reported that alkaloids, phenolic derivatives, and terpenes are among the most potent anti-leishmanial compound classes [16]. Plant derived polyphenols are reported as important sources of *Leishmania* arginase inhibitors [17]. Evidence suggests that arginase is the most investigated enzyme in studies involving flavonoid compounds as enzymatic inhibitors for *Leishmania* [18]. The bioactive compounds of *C. pachystachya* that inhibit *Leishmania* arginase were characterized as glucoside flavonoids [19]. Previous research on the activity of natural compounds as arginase inhibitors provided novel structures that could be used for designing pharmaceutical compounds and might allow a dietary approach to diseases associated with arginase pathway regulation [17,20].

An important part of any treatment with a compound is the selectivity the compound has for its target and the avoidance of toxic and side-effects. Anti-targets are off-targets that a compound would ideally have a specific form of interaction with, in addition to the target [21]. A set of anti-targets that are present in the metabolism of substances has been developed and has found use as an *in silico* protein-ligand interaction assessment [22,23].

In this work, we performed a virtual screening protocol that considers both short-and long-range interactions between interacting molecules. The long-range interactions are characterized by the parameters—the average quasi valence number (AQVN) and the electron-ion interaction potential (EIIP) [24,25]. First, the EIIP/AQVN filter was applied for *in silico* screening of the MetIDB database for anti-leishmanial arginase inhibitors and then followed by 3D QSAR. The database of flavonoid compounds was then filtered and assessed for its binding interactions with *Leishmania* arginase, as well as against human arginase and a battery of anti-targets, in order to select and profile the final set of flavonoids with desired features that may help in the discovery and development of compounds to treat leishmaniasis.

## 2. Results

### 2.1. EIIP/AQVN Filter

**Figure 1:**
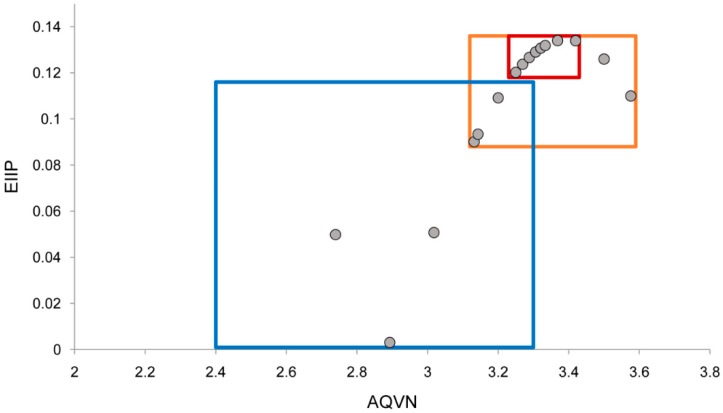
Schematic representation of the EIIP/AQVN criterion for selection of candidate *Leishmania* arginase inhibitors. Extended active group (orange): AQVN (3.13–3.58), EIIP (0.09–0.134). Core active group domain (red): AQVN (3.25–3.42), EIIP (0.12–0.134). Chemical space (blue) AQVN (2.40–3.30) EIIP (0.000–0.116) EIIP/AQVN domain of homologous distribution of >90% compounds from the PubChem Compound Database [30]. Statistics: Extended active group—inside the active wider domain: 21 (87.5%), outside the active wider domain: 3 (12.5%); Core active group—inside the active domain: 12 (50%), outside the active domain: 12 (50%).

The virtual screening (VS) protocol in this paper was based on the application of sequential filters to select candidate anti-leishmanial arginase inhibitors. Previously it was shown for targets in different infectious diseases (HIV, Ebola virus, malaria, bacterial infections) that small molecules with similar AQVN and EIIP values interact with the common therapeutic target [24,25,26]. This resulted in establishing criteria for virtual screening of molecular libraries for compounds with similar therapeutic properties [24,27]. In the series of analysis to be reported here, first, we selected the training set encompassing 24 anti-leishmanial arginase inhibitors from the ChEMBL Target Report Card of *Leishmania* *amazonensis* arginase inhibitors (https://www.ebi.ac.uk/chembl/target/inspect/CHEMBL3108635) [28]. 18 of those entries had IC_50_ values that were used for the building of QSAR models. Out of all the compounds presented in Figure 1, 21 of them are present inside the extended active domain (representing 87.5% of the total) with AQVN and EIIP values within intervals of (3.13–3.58) and (0.09–0.134), respectively. A core group domain was selected with AQVN and EIIP values within the intervals of (3.25–3.42) and (0.12–0.134), respectively, as a criterion for the selection of compounds representing candidate anti-leishmanial arginase inhibitors. By applying EIIP/AQVN-based virtual screening using this criterion, 200 flavonoids were chosen out of the total 5679 compounds from the MetIDB database (www.metidb.org) [29] as candidates for anti-leishmanial arginase inhibitors.

In the previously reported EIIP/AQVN distribution of compounds from the PubChem database (http://www.ncbi.nlm.nih.gov/pccompound) it has been found that 92.5% of compounds are homogenously distributed within EIIP and AQVN intervals (0.00–0.11 Ry) and (2.4–3.3), respectively [25]. This domain of the EIIP/AQVN space, encompassing the majority of known chemical compounds was marked as a “basic EIIP/AQVN chemical space” (BCS). The majority of the core active group of candidate anti-leishmanial arginase inhibitors belongs to the rare chemical compounds that are out of BCS. The proposed EIIP/AQVN VS criteria suggest that testing only a fraction of the compounds belonging to the core active group EIIP/AQVN domain out of a large set of available molecules has a higher chance of being effective against leishmanial arginase than testing compounds with any EIIP/AQVN values. The specificity of the proposed criterion for the selection of compounds representing candidate anti-leishmanial arginase inhibitors is reflected in the fact that this part of AQVN/EIIP space contains only 3% of all chemicals.

### 2.2. 3D-QSAR Model Building

**Figure 2:**
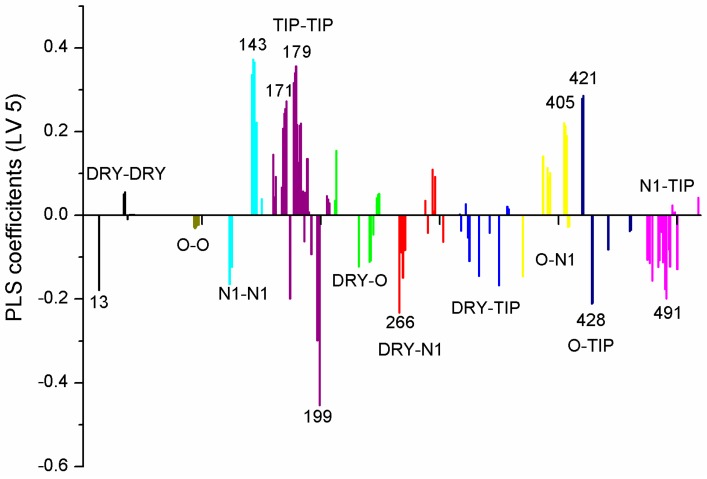
PLS coefficients in the *Leishmania* arginase inhibitors’ QSAR model.

**Figure 3:**
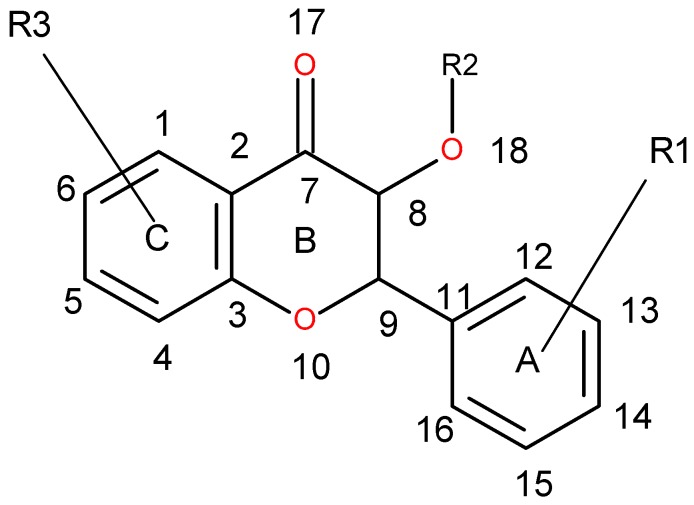
Basic skeleton of known *Leishmania* arginase inhibitors from the ChEMBL database. R1, R3 = OH, OAc; R2 = OAc or hexopyranose ring (see Table S6).

Ten partial least squares (PLS) variables in the arginase inhibitors’ model represent the most significant molecular properties for activity, where there is a negative contribution of variables 13, 199, 266, 428, 491, and positive contributions originating from variables 143, 171, 179, 266, 405, and 421 (Table 1, Figure 2 and Figure 3). Since *R*^2^ values peaked at LV3, a model using three latent variables was used. Tests using scrambled data further validated the model since they had a large influence on the quality of the PLS model, resulting in much lower *R*^2^ values and negative *Q*^2a^ values. Also, fractional factorial design (FFD) convergence was achieved with a much lower number of variables. The tests are shown in the Tables S1–S5.

**Figure 4:**
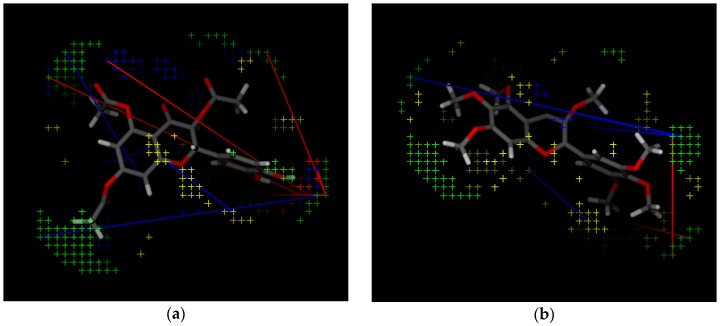
(**a**) The most active compound (CHEMBL3109443); and (**b**) the least active compound (CHEMBL3109440) in the *Leishmania* arginase model with PLS variables. Green dots: TIP probes, yellow dots: DRY probes, blue dots: N1, red dots: O probes. Lines that connect dots are variables, such as DRY—DRY, TIP—TIP, N1—TIP *etc.*

Variable (Var) 13 is related to the distance between two aromatic ring probes; its negative contribution by a relative small distance suggests the presence of two close hydrophobic aminoacid residues (or pocket), which form aromatic interactions with the molecule, and any deviation from the ideal distance contributes negatively to the binding energy and thus, to the experimental activity. Var 266 refers to the distance between aromatic ring and hydrogen bond acceptor, and its negative contribution on such a short distance indicates the presence of a hydrogen bond donor aminoacid residue close to a hydrophobic one. Due to the particularities of hydrogen bonding, small deviations of distance and bond angles negatively affect the energy of interaction. Var 405 corresponds to the distance between hydrogen bond acceptor and donor atoms on counter sides of a molecule. The longer this distance, the higher the activity for such compound, which indicates there is a wide binding site in the receptor. Var 199 corresponds to the curvature of a molecule; the negative contribution is due to steric effects of protein—ligand interactions. Var 428 represents the curved surface point of molecule and its nearest hydrogen bond donor, whose effect is also steric. Var 143 represents the distance between two hydrogen bond donors on counter sides of the molecule. Its physicochemical meaning is similar to Var 405. Var 421, contrary to Var 428, has a positive contribution for the distance between hydrogen bond donor and point on the curved surface of the same atom, and is correlated with activity. Var 419 has a similar physicochemical effect as Var 428. Var 171 and 179 are related to the curvature of two close hydrogen bond donor (OH) groups and therefore, provide a positive contribution to experimental activity. Some of the variables are presented on Figure 4a,b, and shown on the least active compound and the most active compound.

Based on these QSAR properties, the activities of selected candidates were calculated and in order to maintain molecular similarity, PLS scores were monitored, and so the compounds with the best predicted pIC_50_ values were selected.

### 2.3. 3D QSAR Filtering

**Figure 5:**
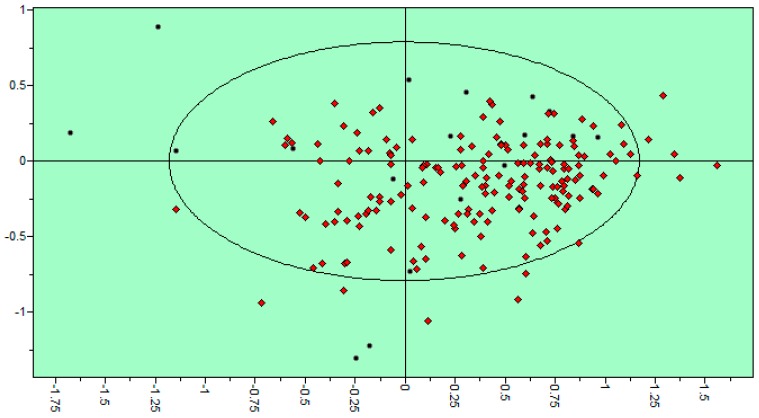
PLS scores of 200 candidates in the *Leishmania* arginase 3D QSAR model. Red: candidates, black: 18 *Leishmania* arginase inhibitors.

The 200 molecules filtered after EIIP criteria were subjected to prediction of their activity using the above described *Leishmania* arginase 3D-QSAR model. The two criteria for selection were: (1) PLS scores around compounds from the model (Figure 5); and (2) best ranking by predicted pIC_50_ values (Table 2). This filtering gave ten candidates which were used for docking into the *Leishmania* arginase structure model, the human arginase structure, and the off-target affinity calculations (see Table S7).

### 2.4. Arginase Docking

Ten compounds were docked into the *Leishmania* arginase model structure, and their binding energies and interactions are presented in Table 2. From the binding energy values, the best three proposed compounds are **13**, **39**, and **22**. Regarding the protein-ligand interactions formed, the binding pattern shows very good agreement to the PLS model. Most compounds had stronger interactions with the *Leishmania* arginase than to the human arginase. The only exceptions were **8** and **59** for Glide; **42** and **50** for Vina; **38**, **39,** and **50** for Autodock 4, and **13** and **38** for Molecular Mechanics/Generalized Born Solvent Surface Area (MM/GBSA). The consensus using all four values gave all compounds except **38** having stronger values for *Leishmania* than for human arginase. Based on this, the compounds show predicted moderate specificity.

**Figure 6:**
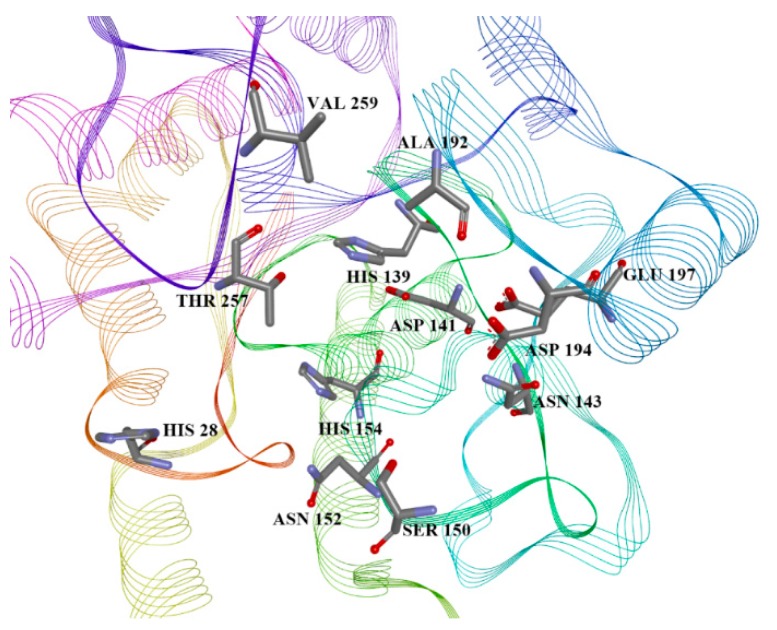
The binding site of *Leishmania* arginase in the homology model, with the most important residues for interaction listed.

Variables can be easily correlated with binding site amino acid positions (Figure 6), as there are only two types of interactions, hydrophilic, originating from Asn, Ser and Thr, and hydrophobic (named “aromatic”), from His, Val and Ala. 

**Figure 7:**
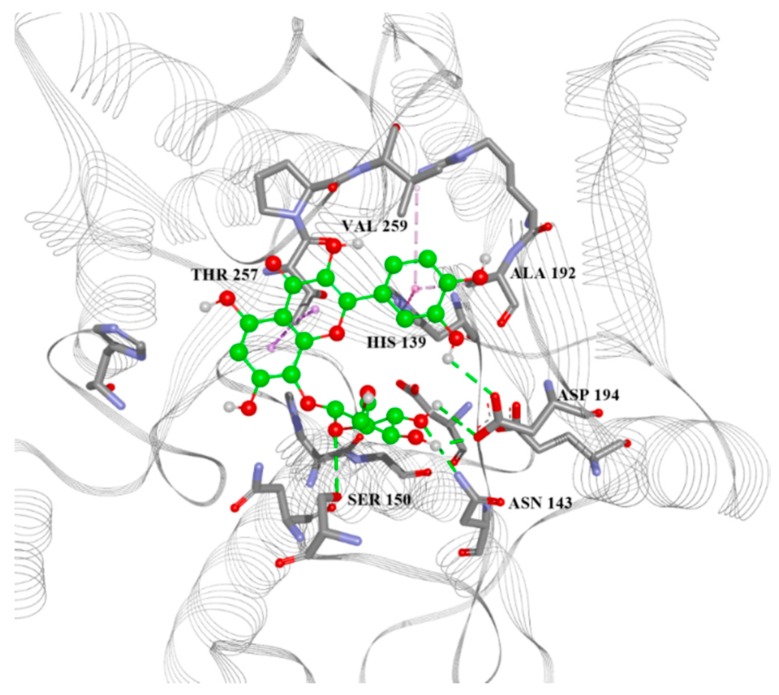
Docking position of best candidate, compound **39** (Table 2) in the binding site of *Leishmania* arginase homology model with marked intermolecular interactions. Green lines: hydrogen bonds, purple: aromatic/hydrophobic interactions

The aminoacid residues shown in Figure 6 are also those used in the binding mode by the known inhibitor 2(*S*)-amino-6-boronohexanoic acid (ABH) in *Leishmania* arginase structure 4iu0, and also interact with compound **39**. The docking pose of the best candidate compound, **39,** is presented in Figure 7.

Pairs of hydrophobic aminoacids correspond to DRY-DRY variables, hydrophobic-hydrophilic to DRY-N1, N1-TIP, O-TIP. Pairs of hydrophilic interactions correspond to N1-N1 and O-N1, while variables TIP-TIP correspond to mixed origins of molecule curvature, either from hydrophobic or hydrophilic groups, and distances from Table 1 correspond to mutual positions of aminoacids in the binding site and the strength of ligand interactions with them, depending on their fit to the binding site shape.

### 2.5. Anti-Target Interaction Matrix

**Figure 8:**
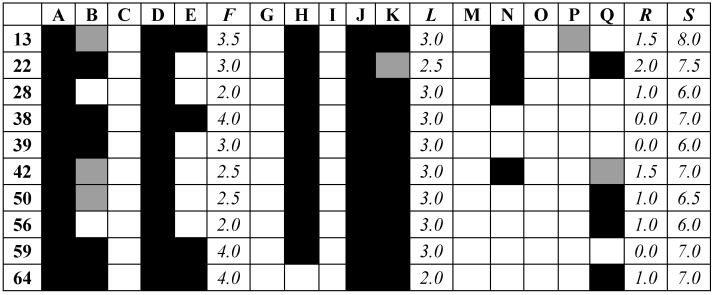
Interaction matrix between proposed ligands and Anti-targets. Color code: black = 1.0; grey = 0.5, white = 0.0. Columns: *A*: Glide XP PXR, *B*: Glide XP SULT, *C*: Glide XP CYP 2a6, *D*: Glide XP CYP 2c9, *E*: Glide XP CYP 3a4, *F*: Glide XP Total, *G*: Autodock 4 PXR, *H*: Autodock 4 SULT, *I*: Autodock 4 CYP 2a6, *J*: Autodock 4 CYP 2c9, *K*: Autodock 4 CYP 3a4, *L*: Autodock 4 Total, *M*: Vina PXR, *N*: Vina SULT, *O*: Vina CYP 2a6, *P*: Vina CYP 2c9, *Q*: Vina CYP 3a4, *R*: Vina Total, *S*: Grand Total.

The top ten compounds from the filtering were then assessed against the anti-target battery. The results of the docking of all the final compounds against the battery of five anti-targets and three docking programs are shown in Figure 8 (full table of docking scores Table S8).

There was broad general agreement between the three docking programs. None of the compounds’ binding score surpassed that of the threshold for CYP P450 2a6, for any docking program, which may be an indication of the relative size of the ligands. There were more interactions found with the anti-targets SULT and CYP P450 3a4. Compound **13** had the highest combined score, while several compounds: **28**, **39**, and **56**, had low interactions; lower than 3.0 for any docking program.

## 3. Discussion

*Leishmanial* arginase, the most examined enzyme in studies involving flavonoid compounds as enzymatic inhibitors for *Leishmania* [18], is essential for the survival of the parasite [11] and is also dissimilar from the mammalian target [10]. Therefore, targeting arginase with flavonoids represents a promising therapeutic strategy for anti-leishmanial treatment. To select flavonoid candidate anti-leishmanial arginase inhibitors, the virtual screening protocol in our study was based on the sequential application of filters. Results of the application of VS based on the EIIP/AQVN approach has shown that the domain encompassing core compounds is highly specific. This part of AQVN/EIIP space contains only 3% of all chemicals. 

One highly ranked flavonoid from our study—*ent*-epicatechin 3-*O*-gallate (compound **13**)—has indeed documented good inhibitory activity on the growth of *Leishmania mexicana* promastigotes using methanol extracts of *Byrsonima crassifolia* bark that contain the referred flavonoid (as reported in KNApSAcK Databases) [31]. The KNApSAcK Family of databases is an Integrated Metabolite–Plant Species Databases with systematically organized relationships between metabolites and their biological origins based on literature data [32]. As the active site in arginase of different strains of *Leishmania* is conserved [10], it could be assumed that the selected candidate inhibitors may have potential to inhibit arginase function in all *Leishmania* strains.

The proposed compounds must also be evaluated for selectivity against human arginase. The anti-target results indicate that the interaction of the proposed candidate compounds with the anti-targets is moderate. It is indeed likely that the desired interaction is precisely a moderate one, neither completely absent (indicating low ability to be metabolized), or too strong and too many interactions that may indicate lability and/or undesired interactions. The flavonoid nature of the compounds may make them ready to be metabolized.

## 4. Materials and Methods

### 4.1. Ligand Data: Sources, Curation, Processing

The collection of compounds was downloaded from the MetIDB database as standard InChI codes [29]. MetIDB is a reference database of experimental and predicted 1H NMR spectra of 6000 flavonoids. Containing a web-based interface, this database provides various interfaces to the data and provides data-mining possibilities. The MetIDB database (www.metidb.org) [29] provides a platform for flavonoids, for their storage and retrieval of 3D chemical structures, 1H NMR spectra, and spectral parameters of fully annotated spectra. The InChI Key was used as a primary key. In addition to the InChI Key, each compound was characterized by InChI Code, chemical formula, exact monoisotopic mass, proton chemical shifts, and where possible, common name and CAS number were also added. The virtual screening protocol reported in this study was based on the application of sequential filters. First we have applied the EIIP/AQVN filter.

### 4.2. EIIP/AQVN Filter

Specific recognition and targeting between interacting biological molecules at distances > 5 Å are determined by the average AQVN and the EIIP [33] derived from the general model pseudopotential [34]:

EIIP = 0.25 Z* sin(1.04 π Z*)(1)

where Z* is the average quasi-valence number (AQVN) determined by:

Z* = ∑*^m^*(*n_i_ Z_i_*/*N*) (2)

where *Z_i_* is the valence number of the *i*th atomic component, *n_i_* is the number of atoms of the *i*th component, *m* is the number of atomic components in the molecule, and *N* is the total number of atoms. EIIP values are calculated according to Equations (1) and (2) and are expressed in Rydberg units (Ry).

Among 3300 currently used molecular descriptors, AQVN and EIIP represent the unique physical properties characterizing the long-range interactions between biological molecules [33]. A strong connection has been demonstrated between the EIIP and AQVN of organic molecules and their biological activity (mutagenicity, carcinogenicity, toxicity, antibiotic and cytostatic activity, *etc.*) [35].

### 4.3. 3D QSAR

In order to build a 3D QSAR model for compound activity prediction, 18 compounds with IC_50_ activities against *Leishmania amazonensis* targeting arginase (Target ID CHEMBL3108635) were downloaded from the ChEMBL database (Table S1) [28]. All compounds were converted to sdf format and then imported. Compounds were imported into the Pentacle QSAR software [36], protonated at pH 7.4, and oriented according to the principal moments of inertia. IC_50_ values were converted to pIC_50_ and used as activities. Standard GRIND descriptors were calculated [36,37], based on molecular interaction field (MIF) probes: DRY (representing hydrophobic interactions), N1 (representing neutral flat NH like in amide, as hydrogen bond donor), O (sp2 carbonyl oxygen, representing hydrogen bond acceptor), TIP (molecular shape descriptor) and the following principal components analysis (PCA) and PLS models were constructed. The number of PC components in the PCA model was five, and in PLS the number of latent variables (LV) was 3. Fractional factorial design (FFD) variables selection was repeated until *R*^2^ and *Q*^2^ values of the PLS model converged. Finally, out of 520 PLS components, 114 were selected after 19 optimizations. *R*^2^_acc_ was 0.99 and *Q*^2^_acc_ was 0.89. Statistics of the PLS model are presented in Table 3. The model was saved for prediction. The EIIP filtered compounds were loaded into the model and their pIC_50_ activities were calculated. Calculations were done in Pentacle 1.05 [36,37,38].

### 4.4. Molecular Docking for Human and Leishmania Arginase

Docking experiments into a *Leishmania amazonensis* arginase model and into a human arginase crystal protein structure were also performed. A homology model of arginase, earlier constructed, was built on a template of the crystal structure of *Leishmania mexicana* arginase in complex with inhibitor ABH (PDBID 4iu0) [39] (obtained from the Protein Model Portal [40], query O96394). Evaluation of the homology model structure using MODELER (https://salilab.org/modeller) resulted in a GA341 score of 1.0, which scores the reliability of a model derived from statistical potentials and predicts it to be reliable when the model score is higher than the pre-specified cutoff of 0.7. A reliable model has a probability of the correct fold >95%, where a fold is correct when at least 30% of its Cα atoms superpose within 3.5 Å of their correct positions. The human arginase protein structure was downloaded from the PDB (2aeb) [39]. Docking was performed with Autodock Vina [41], Glide SP [42], post-processed with MM/GBSA calculations [43], and with Autodock 4 [44]. The grid box was set to span all important residues, according to the previously reported binding site [20]. Water molecules were conserved since they may be important for protein-ligand binding [45,46,47,48,49]

### 4.5. Anti-Target Set

The anti-target battery consisted of three different docking programs and their different scores: Glide XP (XP) [40], Autodock 4 (AD4) [42], and Autodock Vina (Vina) [39], and the proteins pregnane-X-receptor (PXR), sulfotransferase (SULT), cytochromes (CYP) P450 2a6, 2c9, and 3a4. Their structures had resolutions better than 2.6 Å and were downloaded from the PDB (r. 1m13, 2a3r, 1z10, 1og5, 1tqn) [39]. The binding score recorded by their co-crystallized ligand was used as the threshold for determining strong interaction, corresponding to −7.7, −6.3, −7.6, −8.7, and −7.5 with XP; −12.5, −7.5, −6.8, −9.4, and −7.5 for AD4; and −10.3, −6, 08.5, −10, and −7.5 for Vina, which corresponded well with other known inhibitors [22,23]. In order to provide a rough estimate of the interaction, the binding scores were converted to interaction codes represented as:

Score = 0.0 if ΔG − ΔGref > 0.5
(3)

Score = 0.5 if |ΔG − ΔGref| ≤ 0.5
(4)

Score = 1.0 if ΔG − ΔGref < − 0.5
(5)

where ΔG_ref_ is the dock score of the protein with co-crystallized active reference ligand, and ΔG is the dock score for a ligand bound to that protein binding site. These interactions were then shaded white, grey, and black, respectively. Their interaction scores can then be summed across each docking program, across all three docking programs, or the average be taken, or the interaction for a series of compounds with specific proteins.

## 5. Conclusions

Natural compounds present an interesting treatment option against leishmaniasis. A series of flavonoid compounds was filtered according to their average qausi-valence number, electron-ion interaction potential, 3D-QSAR with a homology model of *Leishmania* arginase, and docking to this model in addition to human arginase. An inhibitor was recovered among the actives. The ten most promising candidate compounds were assessed in their interaction profile against a series of five proteins as anti-targets involved in the human metabolism of substances. The compounds present favourable interaction profiles with arginase and against the anti-targets that suggest the compounds may be useful for further investigations and may be better starting points than screening compounds at random. The present results will be confirmed by experimental enzyme inhibition studies in further investigations. These will ultimately show whether these flavonoids are active for several strains and specific, and that they can be useful starting points for the design of safe, easily sourceable, small-molecule compounds for treating the neglected tropical disease leishmaniasis and diminish its burden on populations and healthcare systems.

## Figures and Tables

**Table 1:** Ten most significant PLS variables for *Leishmania* arginase inhibitors’ QSAR model.

	Variable Number	Type	Distance	Latent Variable (LV5) Values	Regions (see Figure 3)
1	13	DRY-DRY	5.20–5.60	−0.1793	Distance between two aromatic rings A and C.
2	266	DRY-N1	2.40–2.80	−0.2333	C3 atom from B ring and carbonyl O10 or carbonyl O17 atom.
3	405	O-N1	16.40–16.80	0.2214	Carbonyl oxygen of OAc at position 1, ring C, and OH oxygen atom, position 14, ring A, or C5 ring C-CH_2_OH oxygen in glucose residue R2 , present only in CHEMBL 3109443, 361362 and 250450.
4	199	TIP-TIP	17.20–17.60	−0.4547	C6-C15 methyl groups.
5	428	O-TIP	4.80–5.20	−0.2121	Ring A, distance between CH_3_ group C14 and nearest OH group C15.
6	143	N1-N1	15.60–16.00	0.3729	Distance between oxygens on A and C rings C14–C5.
7	421	O-TIP	2.00–2.40	0.2867	Distance between H atom and O curvature on the same OH group on ring A C2 position.
8	491	N1-TIP	9.20–9.60	−0.1997	Distance between link O atom, ring B or hexose ring and CH_3_ group curvature on ring A O10–C14.
9	171	TIP-TIP	6.00–6.40	0.2737	Distance between two curvature probes over two neighbor OH groups in ring A C13–C14.
10	179	TIP-TIP	9.20–9.60	0.3568	Distance between two curvature probes over two neighbor OH groups in ring C C6–C4.

**Table 2:** List of docked compounds into *Leishmania* arginase homology model (L.A.) and human arginase crystal structure (H.A.) with QSAR calculated pIC_50_ values for L.A., docking and MM/GBSA energies, and list of interacting aminoacid residues with L.A.

ID	Predicted L.A. pIC_50_	L.A. Vina, Glide, and Autodock 4 Scores, MM/GBSA Energy (kcal/mol)	List of Aminoacid Residues Included in Interactions	H.A. Vina, Glide, and Autodock 4 Scores, MM/GBSA Energy (kcal/mol)
**13**	5.777	−7.6, −5.53, −4.88, −30.80	His28(H), Thr257(A), Asn143(H), Asp141(H), His139(A), Ala192(A)	−6.4, −4.40, −4.77, −32.18
**22**	5.667	−6.8, N/A, −4.51, N/A	Asn143(H), Asp141(H), His139(A), Val259(A)	−6.5, −4.83, −3.82, −36.08
**28**	5.167	−6.6, −5.16, −4.75, −32.15	Asn143(H), His139(A), Thr257(A)	−6.0, −4.07, −3.98, −31.69
**38**	5.054	−7.0, −5.14, −4.01, −35.12	Asp141(H), Asn152(H), Thr257(A), Asp194(H)	−6.9, −5.35, −4.60, −38.47
**39**	5.465	−6.8, −5.23, −4.59, −39.11	Ser150(H), Asn143(H), Asp194(H), Ala192(A), Thr257(A)	−6.5, −4.09, −5.19, −24.99
**42**	5.738	−6.6, −5.61, −5.08, −36.63	His139(A), Asn152(H), Thr257(A)	−6.8, −3.84, −4.01, −32.88
**50**	5.122	−6.3, −5.04, −4.32, −49.23	Ser150(H), Asn152(H), His139(A), Val259(A), Ala192(A)	−7.2, −3.96, −4.35, −18.30
**56**	5.283	−6.6, −5.64, −4.32, −35.87	Asn152(H), His154(A), His139(A), Asp141(H), Val259(A)	−6.0, −5.38, −4.15, −33.92
**59**	5.204	−7, −4.88, −5.59, −32.81	Asn143(H), Glu197(H), Thr257(A), His139(A)	−6.7, −5.61, −4.44, −32.44
**64**	5.673	−6.7, −4.04, −4.39, −40.30	Ser150(H), His139(A), Ala192(A), Val259(A)	−6.0, N/A, −3.89, N/A

**Table 3:** Statistics of PLS model for *Leishmania amazonesis* arginase. Legend: SSX—X variable explanation; SSX_acc_—X accumulation; SDEC—Standard Deviation of Error of Calculation, SDEP—Standard Deviation of Error of Prediction, *R*^2^_acc_—*R*^2^ accumulation; *Q*^2^_acc_—*Q*^2^ accumulation.

Number of Latent Variables	SSX	SSX_acc_	SDEC	SDEP	*R*^2^	*R*^2^_acc_	*Q*^2^_acc_
1	28.31	28.31	0.45	0.61	0.64	0.64	0.34
2	12.94	41.25	0.13	0.33	0.33	0.97	0.81
3	7.95	49.20	0.08	0.25	0.02	0.99	0.89

## Supplementary Materials

Supplementary materials can be accessed at: http://www.mdpi.com/1420-3049/21/5/589/s1.

## Abbreviations

The following abbreviations are used in this manuscript:

HIV: Human immunodeficiency virus
NTD: Neglected tropical disease
PA: polyamines
AQVN: average quasi valence number
EIIP: electron-ion interaction potential
VS: virtual screening
PLS: partial least squares
Var: Variable
CYP: cytochrome
SULT: sulfotransferase
PXR: pregnane-X-receptor
PCA: principal components analysis
XP: Glide XP
AD4: AutoDock 4
Vina: AutoDock Vina
PDB: Protein DataBank
MM/GBSA: Molecular Mechanics/Generalized Born Solvent Surface Area
MIF: Molecular Interaction Fields

## References

[1] Centers for Disease Control and Prevention “Parasites: Leishmaniasis”. http://www.cdc.gov/parasites/leishmaniasis.

[2] Gradoni L. (2013). Epidemiological surveillance of leishmaniasis in the European Union: Operational and research challenges. Euro Surveill..

[3] WHO (2015). Leishmaniasis: Situation and Trends. http://www.who.int/gho/neglected_diseases/leishmaniasis/en/.

[4] Alvar J., Vélez I.D., Bern C., Herrero M., Desjeux P., Cano J., Jannin J., den Boer M., WHO Leishmaniasis Control Team (2012). Leishmaniasis worldwide and global estimates of its incidence. PLoS ONE.

[5] World Health Organization “Leishmaniasis Fact Sheet”. http://www.who.int/mediacentre/factsheets/fs375/en/.

[6] Rogers M., Kropf P., Choi B.S., Dillon R., Podinovskaia M., Bates P., Müller I. (2009). Proteophosophoglycans regurgitated by *Leishmania*-infected sand flies target the l-arginine metabolism of host macrophages to promote parasite survival. PLoS Pathog..

[7] Da Silva M.F., Floeter-Winter L.M. (2014). Arginase in *Leishmania*. Subcell. Biochem..

[8] Colotti G., Ilari A. (2011). Polyamine metabolism in *Leishmania*: From arginine to trypanothione. Amino Acids.

[9] Reguera R.M., Balaña-Fouce R., Showalter M., Hickerson S., Beverley S.M. (2009). *Leishmania* major lacking arginase (ARG) are auxotrophic for polyamines but retain infectivity to susceptible BALB/c mice. Mol. Biochem. Parasitol..

[10] da Silva E.R., Castilho T.M., Pioker F.C., Tomich de Paula Silva C.H., Floeter-Winter L.M. (2002). Genomic organisation and transcription characterisation of the gene encoding *Leishmania* (Leishmania) *amazonensis* arginase and its protein structure prediction. Int. J. Parasitol..

[11] Chawla B., Madhubala R. (2010). Drug targets in *Leishmania*. J. Parasit. Dis..

[12] Machado-Silva A., Guimarães P.P., Tavares C.A., Sinisterra R.D. (2015). New perspectives for leishmaniasis chemotherapy over current anti-leishmanial drugs: A patent landscape. Expert Opin. Ther. Pat..

[13] Rajasekaran R., Chen Y.P. (2015). Potential therapeutic targets and the role of technology in developing novel antileishmanial drugs. Drug Discov. Today.

[14] Fabricant D.S., Farnsworth N.R. (2001). The value of plants used in traditional medicine for drug discovery. Environ. Health Perspect..

[15] Williams C., Espinosa O.A., Montenegro H., Cubilla L., Capson T.L., Ortega-Barría E., Romero L.I. (2003). Hydrosoluble formazan XTT: Its application to natural products drug discovery for *Leishmania*. J. Microbiol. Methods.

[16] Hussain H., Al-Harrasi A., Al-Rawahi A., Green I.R., Gibbons S. (2014). Fruitful decade for antileishmanial compounds from 2002 to late 2011. Chem. Rev..

[17] Girard-Thernier C., Pham T.N., Demougeot C. (2015). The Promise of Plant-Derived Substances as Inhibitors of Arginase. Mini Rev. Med. Chem..

[18] Scotti L., Ishiki H., Mendonça Júnior F.J., Da Silva M.S., Scotti M.T. (2015). In-silico analyses of natural products on *leishmania* enzyme targets. Mini Rev. Med. Chem..

[19] Cruz Ede M., da Silva E.R., Maquiaveli Cdo C., Alves E.S., Lucon J.F., dos Reis M.B., de Toledo C.E., Cruz F.G., Vannier-Santos M.A. (2013). Leishmanicidal activity of *Cecropia pachystachya* flavonoids: Arginase inhibition and altered mitochondrial DNA arrangement. Phytochemistry.

[20] Manjolin L.C., dos Reis M.B., Maquiaveli Cdo C., Santos-Filho O.A., da Silva E.R. (2013). Dietary flavonoids fisetin, luteolin and their derived compounds inhibit arginase, a central enzyme in *Leishmania* (Leishmania) *amazonensis* infection. Food Chem..

[21] Vaz R.J., Klabunde T. (2008). Antitargets. Prediction and Prevention of Drug Side Effects. Series: Methods and Principles in Medicinal Chemistry.

[22] García-Sosa A.T., Sild S., Takkis K., Maran U. (2011). Combined approach using ligand efficiency, cross-docking, and antitarget hits for wild-type and drug-resistant Y181C HIV-1 reverse transcriptase. J. Chem. Inf. Model..

[23] García-Sosa A.T., Maran U. (2014). Improving the use of ranking in virtual screening against HIV-1 integrase with triangular numbers and including ligand profiling with Antitargets. J. Chem. Inf. Model..

[24] Veljkovic V., Mouscadet J.F., Veljkovic N., Glisic S., Debyser Z. (2007). Simple criterion for selection of flavonoid compounds with anti-HIV activity. Bioorg. Med. Chem. Lett..

[25] Veljkovic N., Glisic S., Perovic V., Veljkovic V. (2011). The role of long-range intermolecular interactions in discovery of new drugs. Exp. Opin. Drug Disc..

[26] Veljkovic V., Loiseau P.M., Figadere B., Glisic S., Veljkovic N., Perovic V.R., Cavanaugh D.P., Branch D.R. (2015). Virtual screen for repurposing approved and experimental drugs for candidate inhibitors of EBOLA virus infection. F1000Res..

[27] Veljkovic N., Glisic S., Prljic J., Perovic V., Veljkovic V. (2013). Simple and general criterion for “*in silico*” screening of candidate HIV drugs. Curr. Pharm. Biotechnol..

[28] EMBL-EBI ChEMBL. https://www.ebi.ac.uk/chembl/.

[29] Mihaleva V.V., te Beek T.A., van Zimmeren F., Moco S., Laatikainen R., Niemitz M., Korhonen S.P., van Driel M.A., Vervoort J. (2013). MetIDB: A publicly accessible database of predicted and experimental 1H NMR spectra of flavonoids. Anal. Chem..

[30] Veljkovic V., Veljkovic N., Este J., Huther A., Dietrich U. (2007). Application of the EIIP/ISM bioinformatics concept in development of new drugs. Curr. Med. Chem..

[31] Peraza-Sánchez S.R., Cen-Pacheco F., Noh-Chimal A., May-Pat F., Simá-Polanco P., Dumonteil E., García-Miss M.R., Mut-Martín M. (2007). *Leishmanicidal* evaluation of extracts from native plants of the Yucatan peninsula. Fitoterapia.

[32] Afendi F.M., Okada T., Yamazaki M., Hirai-Morita A., Nakamura Y., Nakamura K., Ikeda S., Takahashi H., Altaf-Ul-Amin M., Darusman L.K. (2012). KNApSAcK family databases: Integrated metabolite-plant species databases for multifaceted plant research. Plant Cell Physiol..

[33] Veljkovic V. (1980). A Theoretical Approach to Preselection of Carcinogens and Chemical Carcinogenesis.

[34] Veljkovic V., Slavic I. (1972). Simple general-model pseudopotential. Phys. Rev. Lett..

[35] Duran A., Zamora I., Pastor M. (2009). Suitability of GRIND-Based Principal Properties for the Description of Molecular Similarity and Ligand-Based Virtual Screening. J. Chem. Inf. Model..

[36] Duran A., Comesaña G., Pastor M. (2008). Development and validation of AMANDA, a new algorithm for selecting highly relevant regions in molecular interaction fields. J. Chem. Inf. Model..

[37] Pastor M., McLay I., Pickett S., Clementi S. (2000). Grid-Independent descriptors (GRIND): A novel class of alignment-independent three-dimensional molecular descriptors. Med. Chem..

[38] Bohn M.F., Shandilya S.M., Albin J.S., Kouno T., Anderson B.D., McDougle R.M., Carpenter M.A., Rathore A., Evans L., Davis A.N. (2013). Crystal structure of the DNA cytosine deaminase APOBEC3F: The catalytically active and HIV-1 Vif-binding domain. Structure.

[39] Protein Data Bank Research Collaboratory for Structural Bioinformatics. http://www.pdb.org/pdb/home/home.do.

[40] PSI|The Protein Model Portal. http://www.proteinmodelportal.org/.

[41] Trott O., Olson A.J. (2010). Autodock Vina: Improving the speed and accuracy of docking with a new scoring function, efficient optimization, and multithreading. J. Comput. Chem..

[42] (2015). Virtual Screening Workflow.

[43] (2015). Prime MM/GBSA.

[44] Morris G.M., Goodsell D.S., Halliday R.S., Huey R., Hart W.E., Belew R.K., Olson A.J. (1998). Automated docking using a Lamarckian genetic algorithm and an empirical binding free energy function. J. Comput. Chem..

[45] García-Sosa A.T. (2013). Hydration properties of ligands and drugs in protein binding sites: Tightly-bound, bridging water molecules and their effects and consequences on molecular design strategies. J. Chem. Inf. Model..

[46] García-Sosa A.T., Mancera R.L. (2010). Free energy calculations of mutations involving a tightly bound water molecule and ligand substitutions in a ligand-protein complex. Mol. Inf..

[47] García-Sosa A.T., Mancera R.L., Dean P.M. (2003). WaterScore: A novel method for distinguishing between bound and displaceable water molecules in the crystal structure of the binding site of protein-ligand complexes. J. Mol. Model..

[48] García-Sosa A.T., Firth-Clark S., Mancera R.L. (2005). Including tightly-bound water molecules in de novo drug design. Exemplification through the *in silico* generation of poly (ADP-ribose) polymerase ligands. J. Chem. Inf. Model..

[49] Lloyd D.G., García-Sosa A.T., Alberts I.L., Todorov N.P., Mancera R.L. (2004). The effect of tightly bound water molecules on the structural interpretation of ligand-derived pharmacophore models. J. Comput. Aid. Mol. Des..

