# Supplementary material for: Arginase Flavonoid Anti-Leishmanial in Silico Inhibitors Flagged against Anti-Targets
Source: Molecules. 2016 May 5;21(5):589. doi: 10.3390/molecules21050589 (PMC6274217; doi:10.3390/molecules21050589)
Supplement: Supplementary file 1 [file molecules-21-00589-s001.pdf]

Supplementary Materials: Arginase Flavonoid Anti-Leishmanial *in Silico* Inhibitors Flagged against Anti-Targets

Sanja Glisic, Milan Sencanski, Vladimir Perovic, Strahinja Stevanovic, Alfonso T. García-Sosa

Table S1. Scrambled data tests.

Original Biodata	Data Scrambling 1	Data Scrambling 2	Data Scrambling 3
6.05	4.91	4.45	4.86
5.8	5.4	6.05	4.06
4.91	3.89	5.8	4.26
4.78	5.8	5.77	5.4
5.43	5.7	5.7	6.05
5.4	4.45	5.68	4.45
5.7	5.68	5.62	5.68
4.45	5.32	5.43	3.89
3.92	6.05	5.4	3.65
5.68	3.65	5.32	4.91
5.32	4.78	4.91	5.77
3.65	5.77	4.86	5.62
4.86	4.86	4.78	4.78
5.77	4.26	4.26	5.43
4.26	4.06	4.06	5.32
5.62	5.62	3.92	5.7
4.06	5.43	3.89	5.8
3.89	3.92	3.65	3.92

Table S2. Data scrambling 1. PLS model—converged at 65 active variables.

Component	SSX	SSXa	SDEC	SDEP	R2	R2a	Q2a
1	26.57	26.57	0.5	0.64	0.56	0.56	0.28
2	17.07	43.64	0.44	0.7	0.1	0.66	0.13
3	14.49	58.13	0.37	0.8	0.1	0.76	−0.14
4	7.98	66.11	0.29	0.85	0.09	0.85	−0.28
5	7.81	73.92	0.25	0.8	0.04	0.89	−0.15

Table S3. Data scrambling 2—35 variables convergence. PLS Model.

Component	SSX	SSXa	SDEC	SDEP	R2	R2a	Q2a
1	35.61	35.61	0.61	0.72	0.34	0.34	0.09
2	24.02	59.64	0.55	0.67	0.12	0.45	0.2
3	10.39	70.03	0.51	0.68	0.09	0.54	0.18
4	7.62	77.65	0.48	0.73	0.05	0.59	0.06
5	8.51	86.16	0.46	0.83	0.03	0.63	−0.23

Table S4. Data scrambling 3—73 variables convergence. PLS Model.

Component	SSX	SSXa	SDEC	SDEP	R2	R2a	Q2a
1	54.85	54.85	0.66	0.76	0.23	0.23	−0.03
2	7.7	62.55	0.22	0.59	0.68	0.91	0.38
3	10.99	73.55	0.16	0.47	0.04	0.95	0.6
4	5.83	79.37	0.13	0.44	0.02	0.97	0.65
5	4.02	83.4	0.11	0.4	0.01	0.98	0.72

True biodata.

Table S5. Statistics of PLS model for *Leishmania amazonesis* Arginase.

Number of latent variables	SSX	SSX_{acc}	SDEC	SDEP	R^2	R^2_{acc}	Q^2_{acc}
1	28.31	28.31	0.45	0.61	0.64	0.64	0.34
2	12.94	41.25	0.13	0.33	0.33	0.97	0.81
3	7.95	49.20	0.08	0.25	0.02	0.99	0.89
4	13.21	62.41	0.05	0.24	0.01	1.00	0.89
5	11.59	74.00	0.04	0.23	0	1.00	0.90

Legend: SSX—X variable explanation; SSX_{acc}—X accumulation; SDEC—Standard Deviation of Error of Calculation, SDEP—Standard Deviation of Error of Prediction, R^2_{acc}—R^2 accumulation; Q^2_{acc}—Q^2 accumulation.

Table S6. Known inhibitors for *Leishmania* arginase from ChEMBL.

Compound Name	Trivial name	Structure	pIC50
CHEMBL3109443	-	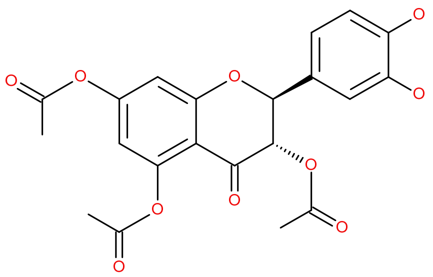	6.05
CHEMBL583912	(−)-EPICATECHIN	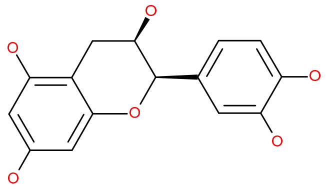	5.8
CHEMBL82242	QUERCITRIN	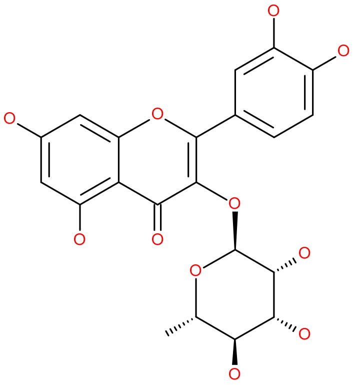	4.91
CHEMBL1078766	Trigonostemone	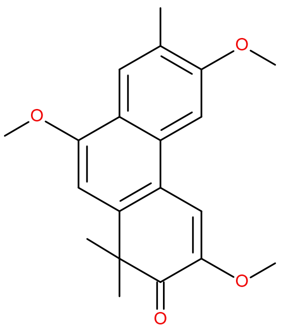	4.78

Table S6. *Cont.*

Compound Name	Trivial name	Structure	pIC50
CHEMBL3109442		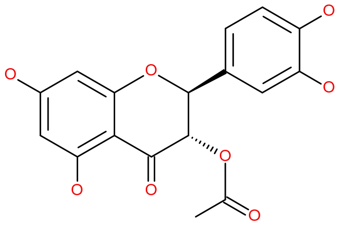	5.43
CHEMBL50	Meletin	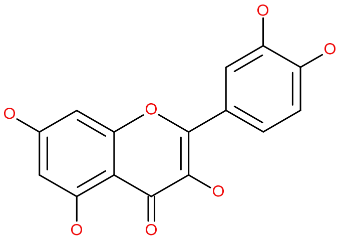	5.4
CHEMBL250450	NSC-407304	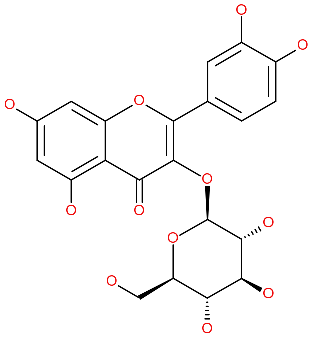	5.7
CHEMBL3109441		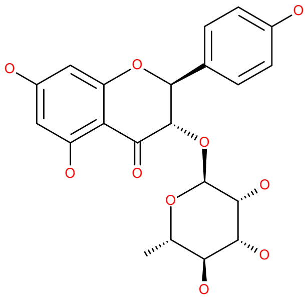	4.45

Table S6. *Cont.*

Compound Name	Trivial name	Structure	pIC50
CHEMBL19074	NSC-115919	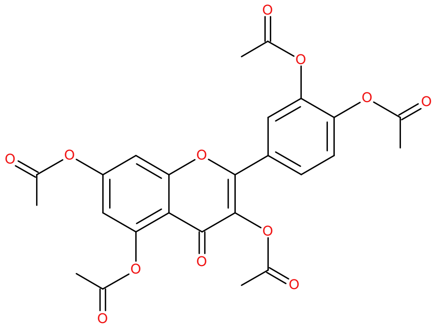	3.92
CHEMBL164	NSC-407290	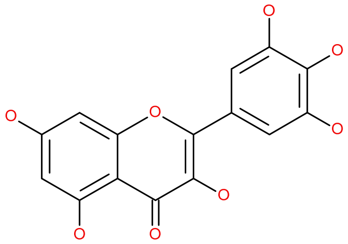	5.68
CHEMBL3109444		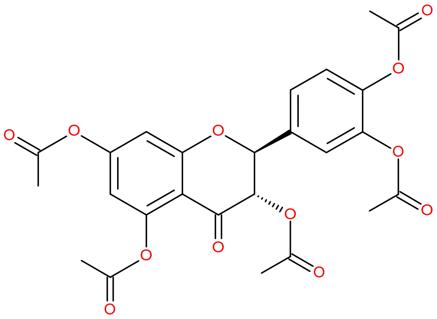	5.32
CHEMBL3109438		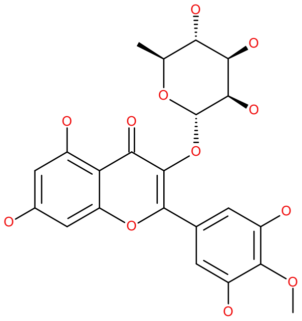	3.65
CHEMBL361362	(+/−)-Syringaresinol	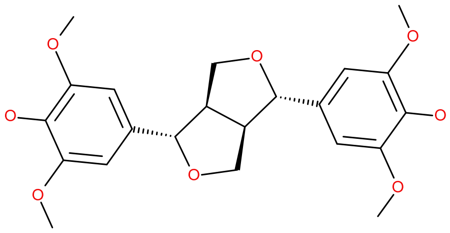	4.86
CHEMBL311498	Cianidol	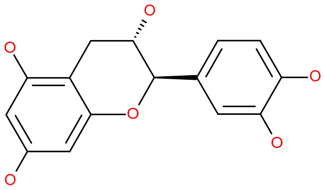	5.77

Table S6. *Cont.*

Compound Name	Trivial name	Structure	pIC50
CHEMBL150	Populnetin	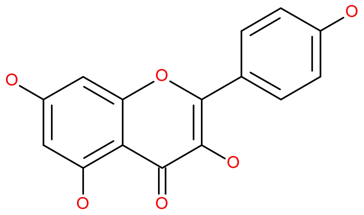	4.26
CHEMBL3109439		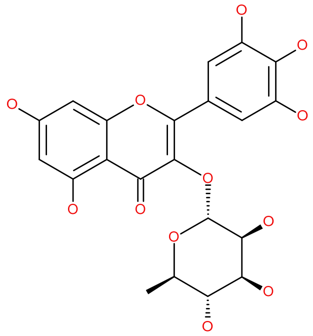	5.62
CHEMBL3109437		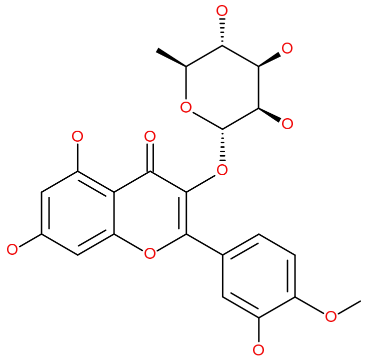	4.06
CHEMBL3109440		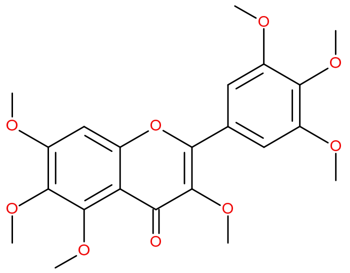	3.89

ChEMBL database compounds used for building of 3D QSAR model.

Table S7. Top ten filtered compounds.

Compound No.	ID	Structure	Predicted pIC_{50}	Name
1	**59**	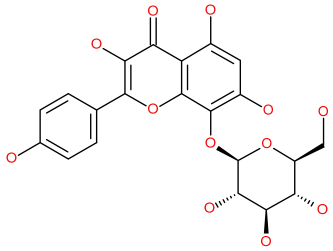	5.204	Herbacetin 8-glucoside
2	**39**	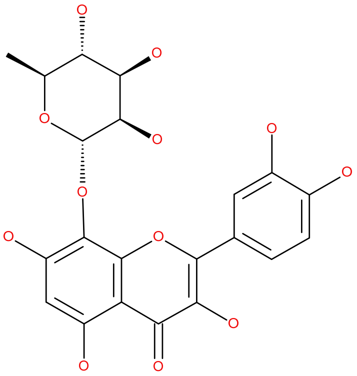	5.465	Gossypetin 8-*O*-rhamnoside CAS 94516-28-6
3	**38**	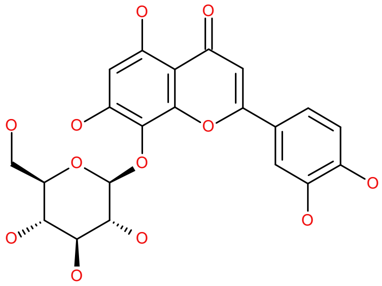	5.054	8-Hydroxyluteolin 8-glucoside
4	**50**	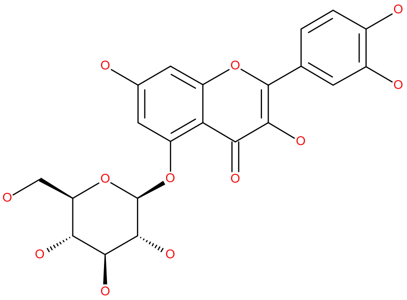	5.122	Quercetin 5-glucoside CAS 34199-21-8
5	**28**	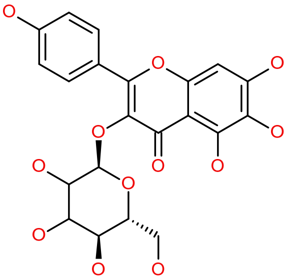	5.167	Scutellarein 3-*O*-glucoside CAS Registry Number 145134-61-8

Table S7. *Cont.*

Compound No.	ID	Structure	Predicted pIC_{50}	Name
6	**56**	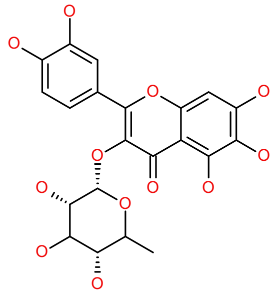	5.283	Quercetagetin 3-*O*-rhamnoside CAS 64543-29-9
7	**64**	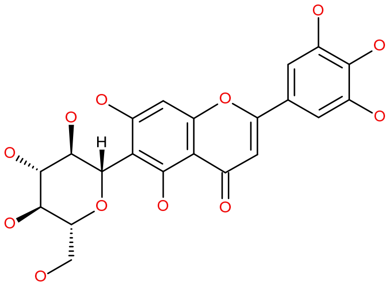	5.673	Isoaffinetin
8	**13**	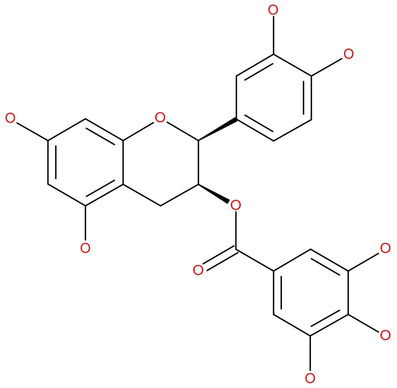	5.777	ent-Epicatechin 3-*O*-gallate CAS 1257-08-5
9	**42**	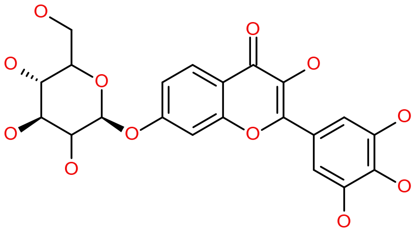	5.738	
10	**22**	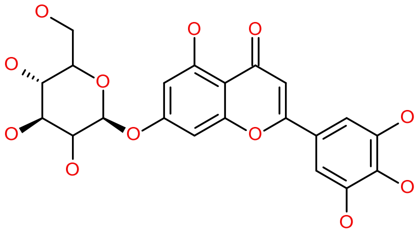	5.667	Tricetin 7-glucoside

Top ten selected candidate compounds from the MetIDB database.

Table S8. Docking values for five anti-target proteins with three different scoring functions/programs.

ID	PXR			SULT			CYP 2a6			CYP 2c9			CYP 3a4		
	glide	ad4	vina	glide	ad4	vina	glide	ad4	vina	glide	ad4	vina	glide	ad4	vina
**13**	−11.992	−9.59	−9.4	−5.88	−11.76	−8	0	26.74	21.3	−13.88	−10.84	−10	−8.84	−11.2	−6.7
**22**	−12.35	−9.79	−8.7	−9.56	−13.28	−9	0	49.88	31.2	−12.78	−11.06	−9.2	0	−7.71	−8.4
**28**	−12.265	−10.27	−8.7	−3.97	−12	−7.1	0	70.31	29.7	−14.08	−11.09	−8.8	0	−10.94	−5
**38**	−12.841	−10.66	−8.7	−9.8	−11.41	−4.8	0	21.4	24.7	−13.95	−11.44	−9.3	−9.09	−10.26	−5.2
**39**	−11.915	−10.39	−9.1	−10.46	−10.82	−5.2	0	47.13	29.1	−12.71	−11.38	−9.2	0	−8.74	−2.1
**42**	−11.646	−9.87	−8.3	−6.52	−12.38	−8.2	0	61.02	24	−11.81	−10.57	−8.7	0	−8.4	−7.4
**50**	−10.523	−9.94	−8.2	−6.36	−11.51	−5.3	0	35.76	20.8	−10.68	−10.52	−9.1	0	−10.82	−6.3
**56**	−12.737	−10.2	−8.6	−4.15	−11.42	−5.1	0	63.75	27.8	−12.4	−10.52	−9.3	0	−9.92	−4.7
**59**	−11.246	−10.2	−8.8	−8.29	−11.31	−5.7	0	34.42	28.5	−12.56	−11.46	−8.8	−10.77	−10.1	−5.3
**64**	−10.733	−9.63	−7.1	−6.91	−8.64	−5.3	0	52.64	26.2	−11.73	−10.36	−8.9	0	−4.84	−7.2

Binding energy score values for the top ten predicted inhibitors with numbers in bold with the five anti-target proteins with three different docking programs. glide: Glide XP; ad4: AutoDock 4; vina: AutoDock Vina; PXR: pregnane-X-receptor; SULT: sulfotransferase; CYP 2a6: cytochrome P450 2a6; CYP 2c9: cytochrome P450 2c9; CYP 3a4: cytochrome P450 3a4.
